# Mindfulness-based cognitive therapy for major depressive disorder- a literature review

**DOI:** 10.1192/j.eurpsy.2021.1320

**Published:** 2021-08-13

**Authors:** D. Vasile, O. Vasiliu, A. Mangalagiu, B. Petrescu, C. Tudor, C. Candea

**Affiliations:** Psychiatry, University Emergency Central Military Hospital Dr. Carol Davila, Bucharest, Romania

**Keywords:** cognitive-behavioral therapy, mindfulness, major depressive disorder, relapse prevention

## Abstract

**Introduction:**

Mindfulness-based cognitive therapy (MBCT) is a third wave cognitive-behavioral therapy (CBT) that incorporates meditation exercises in the classical, structured intervention. Mindfulness has been associated with psychological well-being, and certain symptoms that occur in major depressive disorder (MDD), e.g. worries, ruminations, ideas of incapacity or self-devaluation, are considered potential targets for MBCT.

**Objectives:**

To evaluate the current level of evidence for the MCBT efficacy in MDD.

**Methods:**

A literature serach was performed in the main electronic databases, targeting clinical trials that evaluated in a randomized manner the efficacy of MCBT versus active comparators or placebo in patients with MDD.

**Results:**

MBCT was efficient in a 10-week randomized controlled trial (RCT) versus standard treatment, and it decreased ruminations, increased patients quality of life, mindfulness abilities, and self-compassion. In another randomized, 8-week RCT, MBCT prevented relapses in MDD, with similar rates when compared to psychoeducation and standard treatment. A 26-month follow-up study evidenced the persistence of symptoms improvement detected after 12 months of the trial, when compared to active control group and treatment as usual. MCBT was compared to cognitive therapy in a randomized 8-week trial, and both treatments had similar efficacy in MDD relapse prevention.

**Conclusions:**

MCBT may be an useful adjuvant to the current treatment in acute MDD, but it may also decrease the risk of relapse after psychotherapy termination.

